# The Black and African American Connections to Parkinson’s Disease (BLAAC PD) study protocol

**DOI:** 10.1186/s12883-024-03914-7

**Published:** 2024-10-21

**Authors:** Lana M. Chahine, Naomi Louie, J Solle, Fulya Akçimen, Andrew Ameri, Samantha Augenbraun, Sabrina Avripas, Sarah Breaux, Christopher Causey, Shivika Chandra, Marissa Dean, Elizabeth A. Disbrow, Lauren Fanty, Jessica Fernandez, Erin R. Foster, Erin Furr Stimming, Deborah Hall, Vanessa Hinson, Ashani Johnson-Turbes, Cabell Jonas, Camilla Kilbane, Scott A. Norris, Bao-Tran Nguyen, Mahesh Padmanaban, Kimberly Paquette, Carly Parry, Natalia Pessoa Rocha, Ashley Rawls, Ejaz A. Shamim, Lisa M. Shulman, Rebeka Sipma, Julia Staisch, Rami Traurig, Rainer von Coelln, Peter Wild Crea, Tao Xie, Zih-Hua Fang, Alyssa O’Grady, Catherine M. Kopil, Maggie McGuire Kuhl, Andrew Singleton, Cornelis Blauwendraat, Sara Bandres-Ciga, the BLAAC PD Study and the Global Parkinson’s Genetics Program (GP2), Lana M. Chahine, Naomi Louie, J Solle, Fulya Akçimen, Andrew Ameri, Samantha Augenbraun, Sabrina Avripas, Sarah Breaux, Christopher Causey, Shivika Chandra, Elizabeth A. Disbrow, Lauren Fanty, Jessica Fernandez, Erin R. Foster, Erin Furr Stimming, Deborah Hall, Vanessa Hinson, Ashani Johnson-Turbes, Cabell Jonas, Camilla Kilbane, Scott A. Norris, Bao-Tran Nguyen, Mahesh Padmanaban, Kimberly Paquette, Carly Parry, Natalia Pessoa Rocha, Ashley Rawls, Ejaz A. Shamim, Lisa M. Shulman, Rebeka Sipma, Julia Staisch, Rami Traurig, Rainer von Coelln, Peter Wild Crea, Tao Xie, Alyssa O’Grady, Andrew Singleton, Cornelis Blauwendraat, Sara Bandres-Ciga, Maggie McGuire Kuhl, Catherine M. Kopil, Marissa Dean, Isabel Alfradique-Dunham, Juliana Coleman, Mohamed Elkasaby, Vijayakumar Javalkar, Roger Kelley, David Standaert, Tracy Tholanikunnel, Jamie Toms, Lynae Baskin, Myeshia Bean, Aidan Bonano, Brian Chauppetta, Candace Cromer, Nicolle Crovetto, Kandace Davis, Mariah Delaune, Jennifer Flowers, Randy Foli, Tenisha Franklin, Hanna Guilluly, Christina Griffin, Ashley Hawkins, Jennifer Heliste, Joshua Hines, Jessica Hudson, Nathan Krinickas, Elsa Levenes, Sophia Marathonitis, Crystal Mercado, Maysen Mesaros, James Ryan Parker, Davina Patel, Alexandra Peters, Joseph Richardson, Kyle Rizer, Christina Robinson, Andrea Rosado Chamorro, Marc Rosenbaum, Lauren Ruffrage, Kailey Sajewski, Terrelle Senette, Jenna Smith, Van Smith, Eileen Terrell, Dominique Thomas, Hannah Thomas, Kristin Thompson, Fermine Thomas-Dean, Olga Valdez, Jacqueline Vanegas, Magdaline Volcy, Rebecca Weimer, Mackenzie Williams, Jared Williamson, Dominique Woodhouse, Shayan Abdollah Zadegan, Melissa Kostrzebski, Christi Alessi-Fox, Karen Clark, Debbie Baker, Tanya Parker

**Affiliations:** 1 University of Pittsburgh, 3471 Fifth Avenue, Pittsburgh, PA 15213 USA; 2 The Michael J. Fox Foundation for Parkinson’s Research, New York, NY USA; 3 Laboratory of Neurogenetics, National Institute on Aging, National Institutes of Health, Bethesda, MD USA; 4 Medical University of South Carolina, Charleston, SC USA; 5 NORC at the University of Chicago, Chicago, IL USA; 6 Ochsner Clinic Foundation, New Orleans, LA USA; 7 Louisiana State University Health Sciences Center Shreveport, Shreveport, LA USA; 8 The University of Texas Health Science Center at Houston, Houston, TX USA; 9 University of Alabama at Birmingham, Birmingham, AL USA; 10 University of Florida, Gainesville, FL USA; 11 Washington University in St. Louis, St. Louis, MO USA; 12 Rush University Medical Center, Chicago, IL USA; 13 Kaiser Permanente Mid-Atlantic States, Largo, MD USA; 14 University Hospitals Cleveland Medical Center, Cleveland, OH USA; 15 University of Chicago, Chicago, IL USA; 16 Center for Alzheimer’s Disease and Related Dementias, National Institute on Aging, National Institute of Neurological Disorders and Stroke, National Institutes of Health, Bethesda, MD USA; 17 University of Maryland, Baltimore, MD USA; 18 German Center for Neurodegenerative Diseases (DZNE), Tübingen, Germany; 19 Coalition for Aligning Science, Chevy Chase, MD USA

**Keywords:** Parkinson’s disease, Genetics, Risk, Racial health disparities, African American ancestry, African admixed

## Abstract

Determining the genetic contributions to Parkinson’s disease (PD) across diverse ancestries is a high priority as this work can guide therapeutic development in a global setting. The genetics of PD spans the etiological risk spectrum, from rare, highly deleterious variants linked to monogenic forms with Mendelian patterns of inheritance, to common variation involved in sporadic disease. A major limitation in PD genomics research is lack of racial and ethnic diversity. Enrollment disparities have detrimental consequences on the generalizability of results and exacerbate existing inequities in care. The Black and African American Connections to Parkinson’s Disease (BLAAC PD) study is part of the Global Parkinson’s Genetics Program, supported by the Aligning Science Across Parkinson’s initiative. The goal of the study is to investigate the genetic architecture underlying PD risk and progression in the Black and/or African American populations. This cross-sectional multicenter study in the United States has a recruitment target of up to 2,000 individuals with PD and up to 2,000 controls, all of Black and/or African American ancestry. The study design incorporates several strategies to reduce barriers to research participation. The multifaceted recruitment strategy aims to involve individuals with and without PD in various settings, emphasizing community outreach and engagement. The BLAAC PD study is an important first step toward informing understanding of the genetics of PD in a more diverse population.

## Background

Parkinson’s disease (PD) is the second most common neurodegenerative disorder, affecting 11.8 million people worldwide [1], and its prevalence is expected to increase as the population ages. While there are effective symptomatic therapies [2], there are currently no proven treatments to slow its progression. As a result, the disease progresses inexorably in the majority of patients, leading to a significant burden on patients and their families, disability, and high healthcare costs [1, 3, 4]. A better understanding of the molecular basis of PD is necessary to develop effective disease-modifying therapies and design improved prediction models. Therein, determining the genetic contributions to PD is a high priority.

The etiology of PD relies on an interplay between genetics, environmental and stochastic factors. Monogenic forms are relatively rare, constituting approximately 5% of cases [5]. However, it is estimated that 15–40% of individuals with PD carry pathogenic variants with incomplete penetrance. To date, over 20 genes have been reported to harbor potential disease-causing mutations linked to PD, although many of these findings still require replication. In addition, over 100 loci increasing PD susceptibility have been identified, enhancing our understanding of the risk spectrum underlying PD etiology and our ability to predict disease [5]. However, a major limitation in PD genomics research is the lack of ancestral diversity [6, 7].

While PD may affect any individual, regardless of ancestry, until recently, studies of the genetic contributions to PD have largely been limited to individuals of European descent. This lack of diversity in research participants has negative implications for the generalizability of findings and worsens existing disparities in healthcare. Based on studies of individuals from predominantly European ancestry, the variant-based heritability of PD has been estimated at 16–36% [5, 8]. Estimates on the heritability in other non-European populations are unknown, but preliminary data indicate they may be higher in some populations [9]. In addition, genetic risk scores calculated in European ancestry samples are about 30% less informative when applied to samples of African ancestry [5, 10, 11]. Most importantly, a lack of diversity in studied samples limits the discovery of genetic risk for PD that may be relevant to non-European populations. For example, a recent meta-analysis of multi-ancestry genome-wide association studies (GWAS) in non-European populations identified novel PD risk loci, including several ancestry-specific risk loci [6, 12, 13]. In the first GWAS of PD in African and African admixed ancestries, a novel and population-specific risk factor at the *GBA1* locus was found to be common in PD patients of African ancestry but had not been previously reported in other populations [14]. These findings highlight the critical importance of studying PD genetics across diverse ancestries.

To address the lack of diversity in PD genetic studies, the Global Parkinson’s Genetics Program (GP2) [15], was established with the support of the Aligning Science Across Parkinson’s (ASAP) initiative [16]. The GP2 aims to broadly diversify the scope of PD genetics and make field-enabling discoveries for mechanistic and clinical research, and therapeutic development [15]. Led by the National Institute on Aging (NIA) and implemented by The Michael J. Fox Foundation (MJFF), the GP2 involves researchers from over 60 countries contributing DNA samples and associated phenotype data from member cohorts. The data are harmonized and made available on a managed access cloud platform that enables computational analysis [17].

Individuals of Black and/or African American ancestry [18] are egregiously under-represented in PD research. Several barriers have been identified as contributing to this problem [19]. Increasing representation of the Black and/or African American population in PD genetics research is a key priority for the GP2, and provides the main impetus of the Black and African Americans Connections to Parkinson’s Disease (BLAAC PD) study [20] A framework [21] for the promotion of diversity, equity, and inclusion in genetics and genomics research emphasizes involvement of a multistakeholder team that includes participants and the communities that will be engaged for the study. Partnership development is integral to the conception, design, and implementation of the the BLAAC PD study, as detailed in this protocol paper.

The overall scientific objective of the BLAAC PD study is to investigate the genetic architecture of PD risk and progression in the Black and/or African American ancestry population in the United States. The study aims to [1] Establish a collaborative network across various healthcare centers to collect data and biosamples from Black and/or African American study participants for genetic studies in PD and make the data available to the research community, [2] Determine the frequency of established and potentially novel PD pathogenic variants in the study sample, [3] Conduct genome-wide assessment of risk variants involving cumulative risk score calculation versus disease status and age at onset and comparison with other populations, and heritability estimation, and [4] Explore trans-ethnic fine-mapping, local ancestry, and admixture mapping to establish analysis pipelines for future larger studies.

## Methods

### Study design

This is a cross-sectional multicenter study.

## Study structure and teams

Several individuals and entities collaborate to implement the BLAAC PD study. The principal investigator, co-investigators, and study advisors work closely with all individuals and entities involved. The BLAAC PD study is funded by ASAP and supported by implementation partner MJFF. NORC at the University of Chicago consults on engagement and recruitment efforts, and provides training and technical assistance to the BLAAC PD study team and sites. The Laboratory of Neurogenetics at the NIA provides biosample storage and management along with performing genotyping, sequencing and data analyses. Database creation and management takes place through NIA. The Clinical Trials Coordination Center (CTCC) department in the Center for Health and Technology at the University of Rochester Medical Center provides site management and data monitoring. Data from the BLAAC PD study are released into the Accelerating Medicines Partnership Parkinson’s Disease Program (AMP PD) [22] Knowledge Platform (see below).

At the initiation of the study, an advisory board meeting was convened, composed of field leaders in PD genetics, study recruitment, and a BLAAC PD control participant. The advisory board issued a list of recommendations (Table 1) which have been incorporated into the study as detailed below.

**Table 1:** BLAAC PD study advisory board recommendations

Recommendation	Details
Establish governance and leadership structure	A steering committee and/or dedicated principal investigator is necessary to ensure the decision making, time, and attention is given to the study.
Establish a patient advisory board	An advisory board is necessary to structure the study with a patient-centered approach, enhance value proposition for participants, learn about and address community needs, etc.
Assess study design/characteristics and modify to be patient centered	Protocol changes, additions, and study design must be patient centered to be most effective and impactful for the community.
Focus on community-specific recruitment and engagement	Evaluate strategies and consider best practices for community engagement, building long-term relationships with the community and considering ways to give back to the community.
Develop effective recruitment materials	Frequently asked questions (FAQs) and other materials should be included that increase participant confidence in the study.
Establish meaningful partnerships with sites and provide meaningful support	Include sites at minority-serving and community-based institutions, provide meaningful support to participating site team (such as grant funding to support full-time equivalent time), incorporate diversity, equity, and inclusion expertise and education in site staffing and training

### Study sites

The primary criteria for site selection are: [1] Highly motivated and diverse site teams, [2] Study site serves ethnically and racially diverse patient population and is located near areas with high population density of Black and/or African American communities, and [3] experience and/or partners experienced in community engagement.

**Fig. 1:**
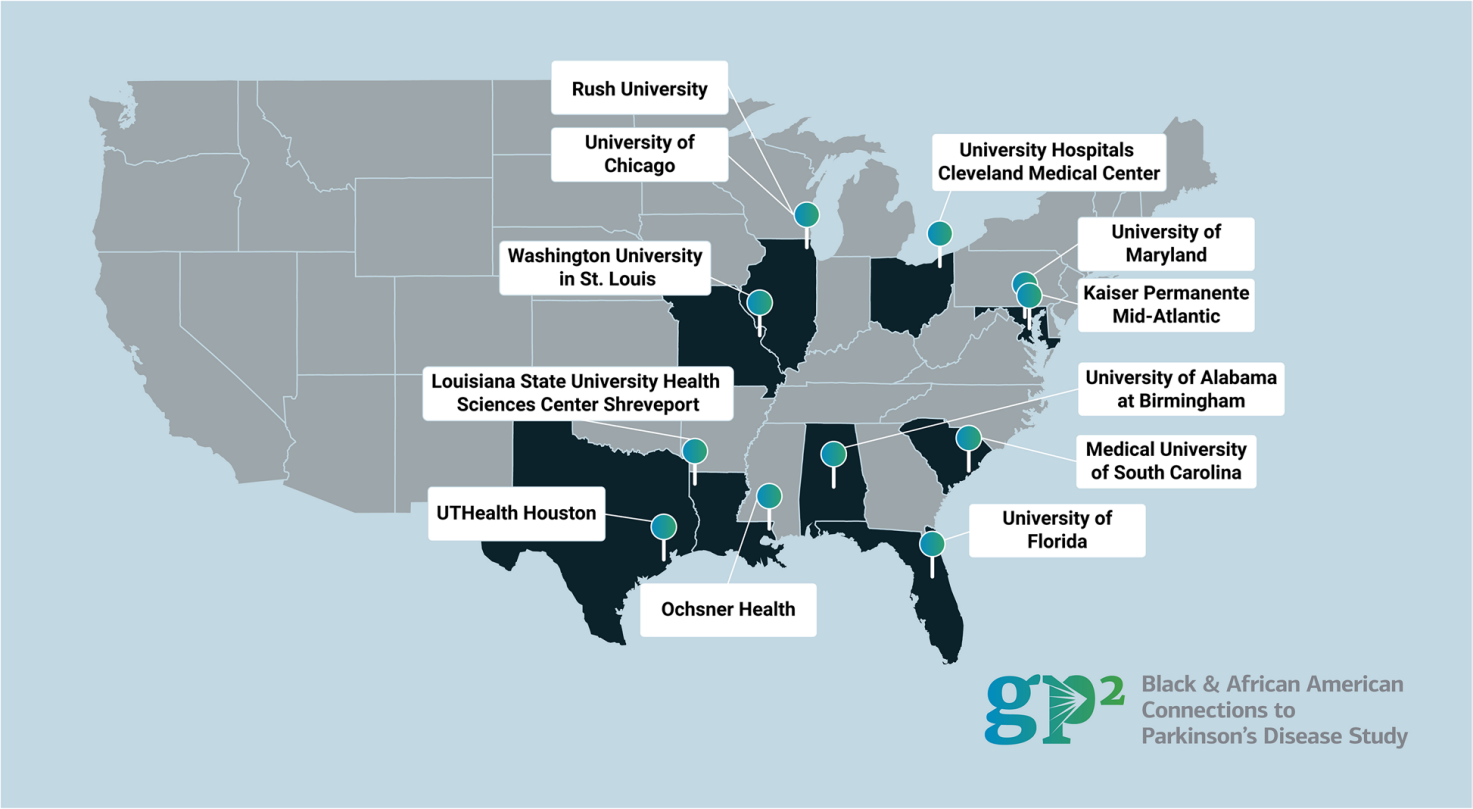
Geographic location of BLAAC PD sites

As of August 2024, there are 11 active study sites from across the United States of America (USA) (Fig. 1). Each site team includes a site investigator and one or more study coordinators, and may include several sub-investigators.

## Study sample

The study aims to recruit a convenience sample of at least 1,000 and up to 2,000 individuals with PD and at least 1,000 and up to 2,000 controls.

### Reducing barriers to research participation

Several barriers to research participation have been identified among individuals with and without PD who identify as Black or African American [23–28]. The BLAAC PD study incorporates several approaches to reduce potential barriers (Table 2) [19, 29].

**Table 2:** BLAAC PD study approaches to reduce barriers to participation in research

Barriers	BLAAC PD Study Approach to Reduce Barriers
**Participant-level**	
Lack of promotion and engagement of the Black and/or African American community with PD research	Sites selected to include centers not conventionally involved in PD multicenter research
	Design and disseminate culturally-responsive study recruitment materials with review and input from site patient advisory boards and local Black and/or African American community members
	Dissemination of education materials about PD in various settings (in-clinic, health fairs, education events, etc.) and in different formats (printed materials (books, brochures, flyers), radio ads, social media)
Fear and mistrust of health care system that has historically and disproportionately harmed Black and African American people through action and/or neglect	Consent form worded to address prior travesties in medical research affecting the community
	Study team training in health equity and how to discuss research protections and processes with potential participants
	Transparency in all aspects of the study
Ambiguity of context and benefit	Consent form outlines significance of studying genetics in diverse populations
	Study protocol includes offering and facilitating participation in another study (29) that provides return of genetic testing results for PD-associated risk genes and genetic counseling
Participant burden/costs	Collect only data that is essential to the study goals and objectives
	Offer option to collect DNA via saliva or other methods as alternative to phlebotomy (weighing risks vs. benefits in light of lower yield of DNA from saliva)
	Offer option of remote collection of consent, data, and specimens
	Home-based and mobile (“research bus”) data collection procedures (select study sites only)
	Cover transportation and childcare costs, and provide remuneration to participants
**Institution-level**	
Underrepresentation at specialized, tertiary care movement disorders centers	Identify study sites that serve diverse and underrepresented populations
	Conduct study assessments in community settings (example: health fairs) (select study sites only)
**Recruitment criteria**	
Restrictive eligibility criteria	Broad inclusion criteria, minimal exclusion criteria for cases and controls (see Table 3)
	Provision of consent for legally authorized representative included in consent form

### Sample size calculations

Power calculations were performed using the GAS power calculation tool by Abecasis et al. [30] Our power to detect an association between common genetic variation (minor allele frequency < 5%) and PD risk was estimated to be 99.8% considering a general disease prevalence = 0.1% [31] and a genotype relative risk effect = 1.5 when including 2,000 cases and 2,000 controls at a significance *p*-value < 5 × 10^−8^, assuming the limitation that the genetic architecture in the Black and/or African American ancestry is unknown.

### Eligibility criteria

The study population is defined as individuals at least 18 years of age in the USA with a clinician-confirmed diagnosis of PD and a control group without a diagnosis of PD or family history of PD or any neurodegenerative condition. Eligibility criteria are shown in Table 3 [32].

**Table 3:** Eligibility criteria for the study

**Inclusion Criteria**	
Cases and controls	Able to provide informed consent
	Aged 18 years or older
	Self-identify as Black or African American
	Proficient in English
Cases only	Meet clinical diagnostic criteria for PD (32)
**Exclusion Criteria**	
Cases and controls	Any condition that, in the investigator’s opinion, precludes the individual’s ability to carry out study activities
Controls only	Diagnosis of PD and/or other neurodegenerative disorder
	Family history of PD and/or neurodegenerative disorder
	Unknown family history of PD and/or other neurodegenerative disorder

### Recruitment strategies

The BLAAC PD study recruitment strategy is multifaceted, multimodal, and evidence-based where data are available [23–28] (Table 4). Sites may implement some or all of these strategies depending on their infrastructure for clinical research and regulatory approvals. These approaches are often layered on general strategies to increase diversity in the clinic population served, such as establishment of local (site-specific) patient advisory boards and utilization of patient navigators. Recruitment materials including postcards and brochures in various formats are available to facilitate recruitment. Recruitment materials are developed in collaboration with a company specializing in design of culturally-informed materials. The content is written by experienced patient recruitment specialists, and reviewed by BLAAC PD study researchers, MJFF communications team, study personnel, BLAAC PD site teams, and participants or representatives from the Black and/or African American community, where possible. Sites are also encouraged to receive input from local patient advisory boards and/or PD support groups. In addition, education materials about PD that could be distributed to various audiences are made available to sites.

**Table 4:** Recruitment strategies employed in the BLAAC PD study

**In-clinic recruitment strategies (cases)**
Disseminate recruitment materials in the clinic and examination rooms
Inform providers at the clinic about the study and provide them with recruitment materials
Identify potential candidates from among patients known to be seen at the site
Prescreen scheduled patient records to identify potential candidates
Partner with providers of potential candidates; “warm handoffs” where possible (direct introduction to study team by the patient’s provider)
Contact patients prior to their scheduled clinic visit by telephone, mail, or through the electronic health record to provide them with information about the study and the opportunity for them to participate
Query the electronic health record for PD international classification of disease codes to identify potential candidates
Query research participant registries to identify potential candidates
**In-clinic recruitment strategies (controls)**
Inform providers in other areas of Neurology clinic about the study and provide them with recruitment materials (to identify disease controls such as patients being seen for headaches)
Recruit spouses or other individuals who accompany the patients (as long as they are not related to the participant)
Query research interest databases to identify potential candidates
Disseminate recruitment materials across the hospital and campus
**Community Outreach (cases)**
Outreach to primary health care providers, community neurologists, nursing homes, and assisted living facilities
Outreach to ancillary healthcare providers and services (physical, occupational, speech therapists, pharmacists, etc.)
Disseminate recruitment materials to patient support groups and patient advocacy groups
Newspaper and radio ads; earned media (local news coverage), social media
Host PD education events in the community (hosted and/or supported by study site and MJFF)
Attend PD education events in the community hosted by other entities (foundations, healthcare systems, others)
Attend PD education events at local and regional medical facilities that serve diverse populations
**Community Outreach (controls)**
Newspaper and radio ads; earned media (local news coverage), social media
Attend health fairs serving diverse populations
Establish relationships with community-based organizations that prioritize health education for diverse populations
Partner with councils of aging; attend social and entertainment events for seniors
Collaborate with other researchers to leverage and share resources

It is expected that the majority of PD cases will be identified from the population of individuals receiving clinical care at the study site, so-called “in-clinic recruitment”. However, outreach to providers who see individuals diagnosed with PD at other local or regional practices and/or medical centers is a critical component of case recruitment. Controls may be recruited in-clinic where possible, for example, if they are accompanying the participant to the clinic and are genetically unrelated to the study participants. However, the primary strategy for recruiting controls is via community outreach and events (Table 4).

## Study procedures

### Ethics

This study is conducted in accordance with the provisions of 21 Code of Federal Regulations Part 50. Written informed consent is obtained by the participant and/or legally authorized representative. The process of informed consent takes place at the beginning of the visit by the site investigator or a delegated study staff member prior to any study procedures.

Institutional review board (IRB) approval has been provided by each of the individual sites’ IRBs and continuing review by the IRB is conducted annually.

### Clinical assessments

A summary of study assessments is shown in Table 5 [33]. Activities may be done in-person or remotely. It is preferred that all activities be completed on the same day but may be completed on separate days. All activities must be performed over the course of up to 30 days. Study assessments consist of two main components: the Minimum Plus Core Data Elements of the GP2 study in addition to BLAAC PD study-specific assessments. Following consent, information is collected about the participant’s sex at birth, age, self-identified race and ethnicity, and education. For cases, confirmation that the participant meets the diagnostic criteria for PD [32] is achieved by interview/examination by the site investigator, review of the patient’s medical history, or input from the treating neurologists as needed. For cases, year of diagnosis, time of symptom onset, whether the participant is receiving levodopa and if so year of initiation are also collected.

**Table 5:** Study assessments for cases and controls

**Activity**	**Cases**	**Controls**
**Informed Consent**	**X**	**X**
**Inclusion/Exclusion**	**X**	**X**
**Demographics**	**X**	**X**
**PD Features**	**X**	
**Clinical Impression of Severity Index for Parkinson’s (CISI-PD) **[33]	**X**	
**Biosample Collection**	**X**	**X**
**Smell Identification Test**	**X**	**X**

Smell Identification Test (SIT): given the strong relationship between olfactory loss and synucleinopathy [34], smell testing is conducted in the BLAAC PD study with the Smell Identification Test Revised™ (Sensonics International, Haddon Heights, NJ; previously known as the University of Pennsylvania Smell Identification Test or UPSIT). The SIT is a 40-item, multiple choice test used to evaluate odor identification. It is a forced-choice “scratch and sniff” test in which subjects must identify an odor among four response alternatives. There are four booklets containing ten odorants each. The total SIT score consists of the number of correct responses out of 40 items. The SIT is collected via self-administration either in-person or remotely. Tests obtained remotely are returned to the site and verified for completeness. The total score is entered into the study database.

## DNA collection and genotyping

**Fig. 2:**
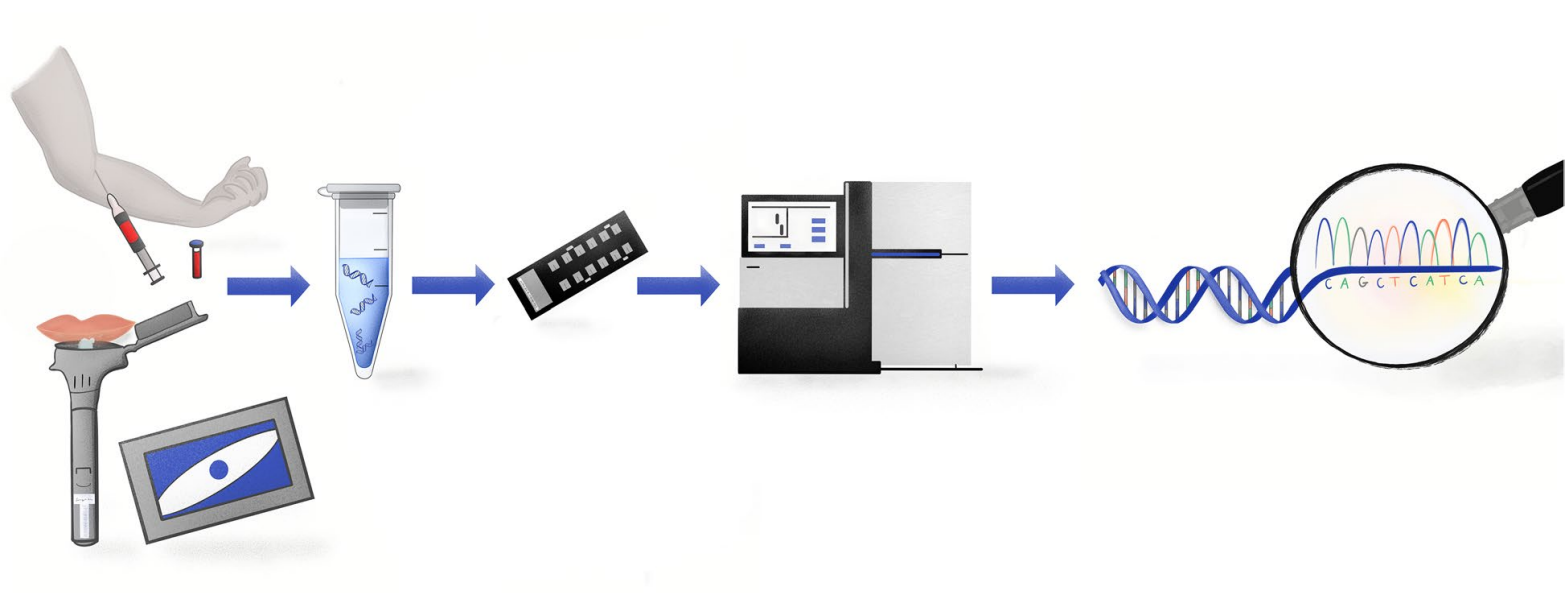
Overview of laboratory procedures. (1) Blood or saliva samples collection (2) DNA isolation (3) Genotyping on NeuroBooster Array (4) Whole-genome sequencing and multiplex ligation-dependent probe amplification (5) Data analyses

An overview of the laboratory protocol is displayed in Fig. 2. DNA collection occurs preferentially via sampling of blood. The DNA sample may be collected via saliva only if the participant is unable or unwilling to donate a blood sample. Blood collection is the preferred method given the higher yield and quality of human DNA as compared to saliva [35, 36].

Blood sample collection: Up to 16mL of blood is drawn by arm venipuncture and collected in EDTA tubes. Blood samples are gently mixed by inverting the tube 8–10 times following collection, labeled, and stored in a freezer at -80 ℃ until shipped.

Saliva sample collection: Saliva is collected using the Oragene-DNA (OG500) DNA Kit (DNA Genotek; Ottowa, Ontario, Canada). Saliva collection kits are provided to sites by the NIA. 2 mL of saliva are collected and mixed according to package instructions. Individuals that participate remotely may be provided a saliva kit in the mail. When occurring remotely, saliva collection is observed by site investigators by way of a HIPAA-compliant video/web conferencing platform. Saliva samples collected via remote self-administration are returned to the study site via a provided shipping kit.

DNA is isolated from blood and from saliva following standard procedures according to manufacturer technical manual and instructions using Maxwell^®^ RSC Whole Blood DNA Kit (Promega Corporation, Madison WI; product #AS1520 and ASB1520) and Maxwell^®^ RSC Stabilized Saliva DNA Kit (Promega Corporation, Madison WI; product # AS1630) respectively.

The molecular genetic analysis is centered on assays of genomic variability including large-scale genotyping and short-read whole-genome sequencing of risk variants and disease-causing mutations, respectively.

Samples are genotyped using the NeuroBooster array (v.1.0, Illumina, San Diego, CA) [37], which includes 1,914,935 variants covering ancestry informative markers, markers for identity by descent determination, and X-chromosome variants for sex determination. Additionally, it incorporates 96,517 customized variants [37].

Raw genotyping data undergoes custom ancestry prediction and pruning using a machine learning method integrated into the GenoTools pipeline [38]. Samples failing to meet inclusion criteria (call rate < 95%, mismatch between genetically determined and clinically reported sex, or excess heterozygosity (|F| statistics > 0.25)) are excluded from further analysis. Ancestry estimates guide sample subsetting, leveraging reference panels from the 1000 Genomes Project, Human Genome Diversity Project, and an Ashkenazi Jewish ancestry dataset.

Following preliminary sample-level quality control, single nucleotide polymorphisms with Hardy-Weinberg Equilibrium (HWE) *p*-value < 1 × 10^−4^ in control samples are removed. Subsequently, variants are pruned based on missingness in case-control status at *P* ≤ 1 × 10^−4^ to eliminate those with non-random missingness, and further pruned for non-random missingness by haplotype at *P* ≤ 1 × 10^−4^. Variants with minor allele frequency (MAF) < 0.005 and HWE *p* < 1 × 10 − ^5^ are filtered out before submission to the TOPMed Imputation Server.  The TOPMed reference panel version r2 includes data from 97,256 reference samples, including over 20,000 African ancestry samples [39], and more than 300 million genetic variants across autosomes 1–22 and the X chromosome. Imputed files are subsequently undergoing pruning with a minor allele count (MAC) threshold of 10 and imputation Rsq of 0.3.

### Whole genome sequencing

Short-read whole-genome sequencing (WGS) is conducted by Psomagen, Inc. In brief, the functional equivalence pipeline [40] from the Broad Institute is used to generate alignments and identify small variants against the GRCh38 reference genome. For sample-level WGS quality control, quality metrics specified by the AMP PD initiative [22] are adhered to. To obtain a set of joint-genotyped variants from samples that pass quality control, the Broad Institute’s joint discovery pipeline is employed, retaining only high-quality variants flagged as “PASS” after recalibration. A call rate > 0.95, genotype quality > 20, read depth > 5, and a heterozygous allele balance between 0.25 and 0.75 are required, as previously described [41]. We also call *GBA1* variants using Gauchian v1.0.2 [42] and genotype known neurological repeat expansions with STRipy v2.240. All pipelines and scripts used are accessible on the GP2 study GitHub [43]. Variants that pass quality control are annotated using ANNOVAR [44]. Compressed Reference-oriented Alignment Map files are visualized using the Integrative Genomics Viewer web browser [45].

### Multiplex ligation-dependent probe amplification

Multiplex ligation-dependent probe amplification (MLPA) is performed using a SALSA MLPA Probemix P051-D1/P052-D2 Parkinson kit (MRC-Holland, Amsterdam, The Netherlands) according to the standard protocols provided by the manufacturer. In summary, SALSA^®^ MLPA^®^ Probemix P052 Parkinson mix 2 detects copy number variations in the *PARK2*,* ATP13A2*,* UCHL1*,* LRRK2*,* GCH1*,* CAV1* and *CAV2* genes, and are used together with SALSA^®^ MLPA^®^ Probemix P051 Parkinson mix 1, which detects copy number variations in *PARK7*,* PINK1 and SNCA*, and offers additional coverage for *ATP13A2* and *PARK2* detection.

Cases are prioritized for MLPA screening based on family history of PD and earlier age of onset. The quantity of DNA is determined using a Qubit fluorometric dsDNA BR assay (#Q33326, Invitrogen, USA). DNA samples are diluted to 12 ng/µL in water, achieving a total of 180 ng of DNA per sample, and 1 µL of 50 mM Tris-HCl buffer at pH 8.5 (#BU-124 S-85, Jena Biosciences, Germany) is used per reaction. PCR fragments are analyzed by capillary electrophoresis on an ABI 3730XL genetic analyzer (Applied Biosystems™, USA) using highly deionized (HiDi) formamide (#4311320, Applied Biosystems™, USA) and GeneScan™ 500 LIZ™ dye size standard (#4322682, Applied Biosystems™, USA).

## Data management

De-identified participant information and data are stored in REDCap [46], a web-based Electronic Data Capture (EDC) database. Data may be collected either on paper forms for entry into the database after collection, or via direct entry into the electronic case report form.

### Data storage and availability

Data from the BLAAC PD study EDC as well as genotyping and sequencing data are included in and accessible through the larger GP2 data repository. The GP2 receives data and DNA samples from the BLAAC PD study and other contributing cohorts from across the globe and releases genetic and other data into the AMP PD Knowledge Platform [22, 47]. AMP PD is managed by MJFF for the NIH and coordinated by multiple organizations including NIH, MJFF and ASAP. Data access is open to qualified researchers worldwide in academia, industry, non-profit organizations and the government.

In addition, the GP2 provides DNA genotyping results back to all cohorts that contribute DNA. Data on AMP PD are available via one of two tiers of access. Tier one allows users to view only summary statistics; tier two provides users with double-pseudonymized, raw level genetic and clinical data. Importantly, all data from the BLAAC PD study and other contributing cohorts are collected under broad study participant consent allowing use of samples and data in future research as per standard GP2 consent guidelines [17, 47].

### Plans for storage of DNA for future use

Leftover DNA following genetic analysis derived from this project are stored in a freezer at the NIA. Samples are stored indefinitely for further analysis as needed, or destroyed if requested by the participant.

## Data analysis plan

To explore genetic risk factors associated with PD in this population through GWAS, imputed dosages (representing genotype probabilities ranging from 0 to 2 for A/A, A/B, or B/B variants, accounting for uncertainty) are analyzed using a logistic regression model. This model adjusts for sex, age, and the first ten principal components (PCs) as covariates. Summary statistics are generated using PLINK 1.9 and 2 [48, 49], and data are filtered for inclusion based on a minimum imputation quality threshold of 0.30 and a MAF > 5%.

To study the influence of genetic variation on disease onset, linear regression will be applied adjusting for similar covariates. Subsequently, cumulative risk score will be evaluated by selecting the risk loci conferring risk for PD across populations. Variants will be weighted by their log odds ratios of population-specific published GWAS [8], giving greater weight to alleles with higher risk estimates, and a composite genetic risk score will be generated across all risk loci. Genetic risk score will be z-transformed prior to analysis, centered on controls, with a mean of zero and a standard deviation of one in the control participants. A binomial generalized linear model will be utilized to evaluate the predictive capability of the polygenic risk score between PD cases and controls, incorporating demographic variables such as age, sex, and the first ten PCs as covariates.

Genome-wide assessment involving GWAS and cumulative risk score calculation versus disease status and age at onset and comparison with other populations will be conducted. When sample size allows, heritability estimates will be calculated implementing Linkage Disequilibrium Score Regression methods (LDSC) [50]. We will be conducting trans-ethnic fine-mapping, local ancestry, and admixture mapping to further dissect the genetic architecture of PD in the context of overall GP2 established analysis pipelines for future larger studies as described elsewhere [13].

To investigate the impact of genetic variation on the age at onset of PD cases, a linear regression model adjusted for the same covariates will be employed. Additionally, linear regression analyses will be conducted to explore the correlation of potential GWAS signals with admixture levels. All analyses are conducted on the Terra platform [51]. GWAS will separately be performed on populations of African and African-admixed ancestries, followed by meta-analyses.

Additionally, in an effort to study the proportion of the phenotype attributable to genetic influence, heritability estimates will be calculated. The narrow-sense heritability (h2), a measure of the additive genetic variance, will be calculated using GREML-LDMS to determine how much of the genetic liability for PD is explained by common genetic variants. This analysis will be adjusted for sex, age, and PCs to account for ascertainment bias. To estimate the influence of rare genetic variation on PD etiology, genome-wide gene-based sequence kernel association test - optimized (SKAT-O) analysis of missense and loss-of-function mutations will be performed to determine the difference in the aggregate burden of rare coding variants between PD cases and controls. Finally, trans-ethnic fine-mapping analyses will be conducted. Additional analyses may include: runs of homozygosity to further study families with recessive patterns of inheritance, and copy number variation through machine learning pipelines using genotyping data.

We will leverage WGS data to screen for rare, highly deleterious protein-altering variants. The data are annotated for protein-coding variation using ancestry-specific databases, including gnomAD v4, and assessed for pathogenicity using established predictors and conservation data across species. Additionally, we will explore potential splicing mechanisms by utilizing Open Targets resources [52]. This comprehensive approach helps identify and characterize variants of interest, providing insights into their potential impact on gene function and disease mechanisms.

MLPA data for copy number variant detection will be analyzed using the Coffalyser.Net™ software package (MRC-Holland, Amsterdam, The Netherlands), according to the provided protocol.

When analyzing SIT scores in the context of PD, a lower score on the test indicates impaired olfactory function. Olfactory function may be designated as abnormal based on available normative data for age and sex [53, 54]. However, the study samples on which normative data were generated lack diversity. Smell test data collected from healthy controls in the BLAAC PD study will be leveraged to improve generalizability of existing normative data to the Black and/or African American population in the USA. From the statistical standpoint, we will explore whether the SIT score is predictive of PD status in the African admixed population by running a logistic regression analysis, specifying PD status as the outcome and SIT score as the primary predictive variable of interest. We will conduct logistic regression adjusted by sex, age, and PCs to account for population stratification (on a normalized scale). In a stepwise manner, we will prune the initial model to build a more parsimonious model. Finally, we will evaluate the overall fit of the model(s) using metrics like AUC, balanced accuracy and pseudo-R-squared to evaluate the predictive strength of the model(s).

## Safety considerations and data/safety monitoring

Questionnaires typically ask participants about the symptoms they are experiencing. For some individuals this can lead to mild distress or boredom. Participants are encouraged to take breaks between tasks, if needed. Risks associated with phlebotomy include pain and bruising at the site where the blood is taken. Sometimes people can feel lightheaded or even faint after having blood drawn.

There is a potential for invasion of privacy or breach in confidentiality. The Laboratory for Neurogenetics at NIA does not receive any personal identifiable information that would link the samples back to individual study participants.

During the course of the study, central monitoring (remote evaluation) is carried out by the CTCC via the web-based EDC. In accordance with ICH Guidelines for Good Clinical Practice (GCP) 5.18, the study is monitored to verify that the rights and well-being of human participants are protected, the reported study data are accurate and complete, and the conduct of the study is in compliance with the currently approved protocol/amendment(s), with GCP, and with the applicable regulatory requirement(s). CTCC conducts remote monitoring of sites’ informed consents, completeness of the EDC questionnaires, and other documents if deemed necessary.

## Discussion

The BLAAC PD study aims to generate phenotypic and genetic data on up to 4,000 individuals with or without PD who are of Black and/or African American ancestry. Genetically, most individuals in the USA who identify as Black or African American have African or African admixed ancestries [55]. While this sample size is modest compared to similar studies of PD risk in European ancestry populations, it is an important first step toward the investigation of genetic contributors to PD in these underserved and underrepresented populations. Several aspects of the study design consider barriers to research participation and aim to mitigate them. The recruitment strategy is multimodal and multifaceted and can be customized according to the site and community within which the study is occurring.

A sufficient sample size is critical towards identifying and validating low-frequency genetic risk variants or rare disease-causing mutations, which may be key contributors to the genetic architecture of disease in a given population. The BLAAC PD study has instituted a variety of innovative recruitment efforts to achieve the target sample size. Given the relatively low prevalence of PD in the general population, population-based approaches to sample identification are expected to be lower yield for identification of cases. For the PD arm of the study, the majority of individuals will thus likely be identified from among the population of individuals receiving clinical care at the site, so-called “in-clinic recruitment”. Given the large volume (thousands) of patients with PD seen at participating sites, this maximizes the likelihood of achieving the target sample size within the recruitment period while also ensuring diagnostic accuracy, given the expertise of providers seeing PD patients in specialized movement disorders clinics. However, substantial efforts are underway to recruit participants receiving care at community-based health clinics , and diagnosis will be confirmed by the study neurologist at the time of the study visit. Community engagement and community-based recruitment strategies aim to identify individuals with PD but are primarily being implemented to recruit controls.

A key aspect of genetic discovery in neurodegenerative disorders is accurate classification of cases and controls. Diagnostic inaccuracy can be high, occurring in over 20% of cases, especially among older adults [56]. However, accuracy of diagnosis improves under subspecialist care [57]. Diagnosis can be further supported by biomarkers, including dopamine transporter imaging [32] in the clinical setting or in the research setting, by in vivo assessment of abnormal alpha-synuclein and other markers of neurodegenerative pathologies [58], many of which can only be reliably measured on cerebrospinal fluid or tissues such as skin.

To mitigate barriers to research participation, the BLAAC PD study prioritizes minimization of participant and site burden in this study. For these and other reasons, imaging, tissue, and biofluid biomarkers are not collected. However, there is confidence in classification of cases and controls in this study, given the expertise of the site investigators and sub-investigators, all of whom have movement disorders expertise. In addition, study assessments include the SIT. Olfactory loss is highly predictive of alpha-synucleinopathy [34] (though with the caveat that most studies that demonstrated this relationship were predominantly composed of White participants). The SIT is included as a study assessment and a surrogate for neurodegenerative alpha-synucleinopathies. In future work, SIT data from BLAAC PD study control participants can contribute to creating SIT normative data that are more generalizable to diverse PD populations. Notably, some genetic traits associated with increased PD risk, such as *LRRK2* and *PRKN* pathogenic variants [34], are associated with lower likelihood of both olfactory loss and underlying alpha-synucleinopathy [59]. Further investigation of this finding in diverse cohorts is needed.

One of the key aspects of advancing personalized medicine is the integration of genetic research findings into information that is useful in clinical practice and on the individual level. In order to provide participants the opportunity to learn information from the study, in real time, that may be of interest or use to them on the individual level, the BLAAC PD study encourages participants with PD to enroll in another study, PD GENEration [29]. PD-GENEration provides genetic counseling and return of genetic testing results for clinically relevant PD-associated risk genes [29]. As the BLAAC PD study and GP2 initiatives continue to produce valuable insights into the genetic factors contributing to PD, it is crucial to emphasize the importance of translating these findings into clinical screening panels. Once the results from these studies are fully replicated and validated, they have the potential to enhance the precision of screening processes, enabling earlier and more accurate diagnosis. The translation from research to clinical application could significantly impact patient outcomes, making it a critical focus for ongoing discussions in the field.

A limitation of the BLAAC PD study design is that it is cross-sectional. A longitudinal study could provide richer data on genetic contributors to disease progression, offering insights into genetic-phenotypic relationships.

The BLAAC PD study, a part of the GP2, aims to investigate the genetic architecture of PD in individuals of Black and/or African American ancestry in the United States. The study develops partnerships with participants and the communities that will be engaged in the research and incorporates several strategies to reduce barriers to participation. The BLAAC PD study will provide foundational insights and valuable data that will drive forward our understanding of the molecular underpinnings of PD.

## Data Availability

No datasets were generated or analysed during the current study.

## Abbreviations

AMP PD: Accelerating Medicines Partnership Parkinson’s disease
ASAP: Aligning Science Across Parkinson’s
AUC: Area under the ROC Curve
BLAAC PD: Black and African American Connections to Parkinson’s Disease
CISI-PD: Clinical Impression of Severity Index for Parkinson’s
CTCC: Clinical Trials Coordination Center
DNA: Deoxyribonucleic acid
EDC: Electronic Data Capture
EDTA: Ethylenediaminetetraacetic acid
FAQ: Frequently Asked Questions
GAS: Genetic Association Study
GCP: Good Clinical Practice
GP2: Global Parkinson’s Genetics Program
GWAS: Genome-wide association study
HIPAA: Health Insurance Portability and Accountability Act
HWE: Hardy-Weinberg Equilibrium
ICH: The International Council for Harmonisation of Technical Requirements for Pharmaceuticals for Human Use
IRB: Institutional Review Board
LDSC: Linkage Disequilibrium Score Regression
LNG: Laboratory for Neurogenetics
MAC: Minor allele count
MAF: Minor allele frequency
MDS: Movement Disorder Society
MJFF: The Michael J. Fox Foundation
MLPA: Multiplex ligation-dependent probe amplification
NIH: National Institutes of Health
NIA: National Institute on Aging
NORC: NORC at the University of Chicago
PCs: Principal components
PD: Parkinson’s disease
SIT: Smell Identification Test
SKAT-O: Sequence kernel association test-optimized
USA: United States of America
UPSIT: University of Pennsylvania Smell Identification Test
WGS: Whole genome sequencing

## References

[1] Global Burden of Disease. Global, regional, and national burden of disorders affecting the nervous system, 1990–2021: a systematic analysis for the global burden of Disease Study 2021. Lancet Neurol. 2024;23(4):344–81. 38493795 10.1016/S1474-4422(24)00038-3 PMC10949203

[2] Foltynie T, Bruno V, Fox S, Kühn AA, Lindop F, Lees AJ. Medical, surgical, and physical treatments for Parkinson’s disease. Lancet. 2024;403(10423):305–24. 38245250 10.1016/S0140-6736(23)01429-0

[3] Yang W, Hamilton JL, Kopil C, Beck JC, Tanner CM, Albin RL, et al. Current and projected future economic burden of Parkinson’s disease in the U.S. Npj Parkinson’s Dis. 2020;6(1):15. 32665974 10.1038/s41531-020-0117-1 PMC7347582

[4] Chaudhuri KR, Azulay JP, Odin P, Lindvall S, Domingos J, Alobaidi A, et al. Economic burden of Parkinson’s disease: a multinational, real-world, cost-of-illness study. Drugs Real World Outcomes. 2024;11(1):1–11. 38193999 10.1007/s40801-023-00410-1 PMC10928026

[5] Blauwendraat C, Nalls MA, Singleton AB. The genetic architecture of Parkinson’s disease. Lancet Neurol. 2020;19(2):170–8. 31521533 10.1016/S1474-4422(19)30287-X PMC8972299

[6] Khani M, Cerquera-Cleves C, Kekenadze M, Wild Crea P, Singleton AB, Bandres-Ciga S. Towards a global view of Parkinson’s Disease genetics. Ann Neurol. 2024;95(5):831–42. 38557965 10.1002/ana.26905 PMC11060911

[7] Schumacher-Schuh AF, Bieger A, Okunoye O, Mok KY, Lim SY, Bardien S, et al. Underrepresented populations in Parkinson’s genetics research: current landscape and future directions. Mov Disord. 2022;37(8):1593–604. 35867623 10.1002/mds.29126 PMC10360137

[8] Nalls MA, Blauwendraat C, Vallerga CL, Heilbron K, Bandres-Ciga S, Chang D, et al. Identification of novel risk loci, causal insights, and heritable risk for Parkinson’s disease: a meta-analysis of genome-wide association studies. Lancet Neurol. 2019;18(12):1091–102. 31701892 10.1016/S1474-4422(19)30320-5 PMC8422160

[9] Loesch DP, Horimoto A, Heilbron K, Sarihan EI, Inca-Martinez M, Mason E, et al. Characterizing the genetic architecture of Parkinson’s disease in latinos. Ann Neurol. 2021;90(3):353–65. 34227697 10.1002/ana.26153 PMC8457043

[10] Martin AR, Gignoux CR, Walters RK, Wojcik GL, Neale BM, Gravel S, et al. Human demographic history impacts genetic risk prediction across diverse populations. Am J Hum Genet. 2017;100(4):635–49. 28366442 10.1016/j.ajhg.2017.03.004 PMC5384097

[11] Saffie Awad P, Makarious MB, Elsayed I, Sanyaolu A, Wild Crea P, Schumacher, Schuh AF et al. Insights into Ancestral Diversity in Parkinsons Disease Risk: A Comparative Assessment of Polygenic Risk Scores. MedRxiv [Preprint]. 2024 May 9:2023.11.28.23299090 10.1038/s41531-025-00967-4 PMC12229533 40610451

[12] Foo JN, Chew EGY, Chung SJ, Peng R, Blauwendraat C, Nalls MA, et al. Identification of risk loci for Parkinson Disease in asians and comparison of risk between asians and europeans: a genome-wide Association study. JAMA Neurol. 2020;77(6):746–54. 32310270 10.1001/jamaneurol.2020.0428 PMC7171584

[13] Kim JJ, Vitale D, Otani DV, Lian MM, Heilbron K, Iwaki H, et al. Multi-ancestry genome-wide association meta-analysis of Parkinson’s disease. Nat Genet. 2024;56(1):27–36. 38155330 10.1038/s41588-023-01584-8 PMC10786718

[14] Rizig M, Bandres-Ciga S, Makarious MB, Ojo OO, Crea PW, Abiodun OV, et al. Identification of genetic risk loci and causal insights associated with Parkinson’s disease in African and African admixed populations: a genome-wide association study. Lancet Neurol. 2023;22(11):1015–25. 37633302 10.1016/S1474-4422(23)00283-1 PMC10593199

[15] GP2. The Global Parkinson’s Genetics Program. Mov Disord. 2021;36(4):842–51. 33513272 10.1002/mds.28494 PMC9290711

[16] Initiative ASAP. https://parkinsonsroadmap.org/#. Accessed 9 Aug 2024.

[17] The Global. Parkinson’s Genetics Program. https://gp2.org/.

[18] Flanagin A, Frey T, Christiansen SL. Updated guidance on the reporting of race and ethnicity in medical and science journals. JAMA. 2021;326(7):621–7. 34402850 10.1001/jama.2021.13304

[19] George S, Duran N, Norris K. A systematic review of barriers and facilitators to minority research participation among African americans, latinos, Asian americans, and Pacific islanders. Am J Public Health. 2014;104(2):e16-31. 24328648 10.2105/AJPH.2013.301706 PMC3935672

[20] Bandres-Ciga S. Black and African American connections to Parkinson’s disease study: addressing missing diversity in Parkinson’s disease genetics. Mov Disord. 2022;37(7):1559–61. 35488798 10.1002/mds.29042 PMC9348590

[21] Rebbeck TR, Bridges JFP, Mack JW, Gray SW, Trent JM, George S, et al. A Framework for promoting diversity, equity, and inclusion in Genetics and Genomics Research. JAMA Health Forum. 2022;3(4):e220603. 35755401 10.1001/jamahealthforum.2022.0603 PMC9223088

[22] Iwaki H, Leonard HL, Makarious MB, Bookman M, Landin B, Vismer D, et al. Accelerating Medicines Partnership: Parkinson’s Disease. Genet Resourc Mov Disord. 2021;36(8):1795–804. 10.1002/mds.28549 PMC8453903 33960523

[23] Natale P, Saglimbene V, Ruospo M, Gonzalez AM, Strippoli GF, Scholes-Robertson N, et al. Transparency, trust and minimizing burden to increase recruitment and retention in trials: a systematic review. J Clin Epidemiol. 2021;134:35–51. 33515656 10.1016/j.jclinepi.2021.01.014

[24] Boden-Albala B, Rebello V, Drum E, Gutierrez D, Smith WR, Whitmer RA, et al. Use of community-engaged research approaches in clinical interventions for neurologic disorders in the United States: a scoping review and future directions for improving health equity research. Neurology. 2023;101(7 Suppl 1):S27-46. 37580148 10.1212/WNL.0000000000207563

[25] Adrissi J, Fleisher J. Moving the Dial toward Equity in Parkinson’s Disease Clinical Research: a review of current literature and future directions in diversifying PD clinical trial participation. Curr Neurol Neurosci Rep. 2022;22(8):475–83. 35713775 10.1007/s11910-022-01212-8 PMC9578442

[26] Dobkin RD, Amondikar N, Kopil C, Caspell-Garcia C, Brown E, Chahine LM, et al. Innovative recruitment strategies to increase diversity of participation in Parkinson’s Disease Research: the Fox Insight Cohort Experience. J Parkinsons Dis. 2020;10(2):665–75. 32250321 10.3233/JPD-191901 PMC7242847

[27] Sanchez AV, Ison JM, Hemley H, Willis A, Siddiqi B, Macklin EA, et al. Designing the fostering inclusivity in Research Engagement for underrepresented populations in Parkinson’s Disease study. Contemp Clin Trials. 2022;115:106713. 35202842 10.1016/j.cct.2022.106713

[28] Vaswani PA, Tropea TF, Dahodwala N. Overcoming barriers to Parkinson Disease Trial Participation: increasing diversity and novel designs for Recruitment and Retention. Neurotherapeutics. 2020;17(4):1724–35. 33150545 10.1007/s13311-020-00960-0 PMC7851248

[29] PD GENEration. Informing people with parkinson’s disease of their gene variant status: PD GENEration, a North American observational and registry study. https://www.parkinson.org/PDGENEration.

[30] Abecasis G. Genetic Association Study (GAS) Power Calculator. https://csg.sph.umich.edu/abecasis/cats/gas_power_calculator/index.html.

[31] Tysnes OB, Storstein A. Epidemiology of Parkinson’s disease. J Neural Transm (Vienna). 2017;124(8):901–5. 28150045 10.1007/s00702-017-1686-y

[32] Postuma RB, Berg D, Stern M, Poewe W, Olanow CW, Oertel W, et al. MDS clinical diagnostic criteria for Parkinson’s disease. Mov Disord: Off J Mov Disord Soc. 2015;30(12):1591–601. 10.1002/mds.26424 26474316

[33] Martínez-Martín P, Forjaz MJ, Cubo E, Frades B, de Pedro Cuesta J. Global versus factor-related impression of severity in Parkinson’s disease: a new clinimetric index (CISI-PD). Mov Disord. 2006;21(2):208–14. 16161158 10.1002/mds.20697

[34] Siderowf A, Concha-Marambio L, Lafontant D-E, Farris CM, Ma Y, Urenia PA, et al. Assessment of heterogeneity and disease onset in the Parkinson’s progression markers Initiative (PPMI) cohort using the α-synuclein seed amplification assay: a cross-sectional study. Lancet Neurol. 2023;22(5):407–17. 37059509 10.1016/S1474-4422(23)00109-6 PMC10627170

[35] Hu Y, Ehli EA, Nelson K, Bohlen K, Lynch C, Huizenga P, et al. Genotyping performance between saliva and blood-derived genomic DNAs on the DMET array: a comparison. PLoS ONE. 2012;7(3):e33968. 22448283 10.1371/journal.pone.0033968 PMC3309006

[36] Samson CA, Whitford W, Snell RG, Jacobsen JC, Lehnert K. Contaminating DNA in human saliva alters the detection of variants from whole genome sequencing. Sci Rep. 2020;10(1):19255. 33159102 10.1038/s41598-020-76022-4 PMC7648094

[37] Bandres-Ciga S, Faghri F, Majounie E, Koretsky MJ, Kim J, Levine KS et al. NeuroBooster array: a genome-wide genotyping platform to study neurological disorders across diverse populations. Mov Disord. 10.1002/mds.29902. Accessed 16 Sept 2024. (online ahead of print). 10.1002/mds.29902 PMC11568947 39283294

[38] Genotools. https://github.com/GP2code/amp-pd-v3-pc-prs.

[39] Kowalski MH, Qian H, Hou Z, Rosen JD, Tapia AL, Shan Y, et al. Use of > 100,000 NHLBI Trans-Omics for Precision Medicine (TOPMed) Consortium whole genome sequences improves imputation quality and detection of rare variant associations in admixed African and Hispanic/Latino populations. PLoS Genet. 2019;15(12):e1008500. 31869403 10.1371/journal.pgen.1008500 PMC6953885

[40] Regier AA, Farjoun Y, Larson DE, Krasheninina O, Kang HM, Howrigan DP, et al. Functional equivalence of genome sequencing analysis pipelines enables harmonized variant calling across human genetics projects. Nat Commun. 2018;9(1):4038. 30279509 10.1038/s41467-018-06159-4 PMC6168605

[41] Lange LM, Avenali M, Ellis M, Illarionova A, Keller Sarmiento IJ, Tan A-H, et al. Global Parkinson’s Genetics Program (GP2) monogenic network protocol: elucidating causative gene variants in hereditary Parkinson’s disease. NPJ Parkinsons Dis. 2023;9(1):100. 37369645 10.1038/s41531-023-00526-9 PMC10300084

[42] Toffoli M, Chen X, Sedlazeck FJ, Lee CY, Mullin S, Higgins A, et al. Comprehensive short and long read sequencing analysis for the Gaucher and Parkinson’s disease-associated GBA gene. Commun Biol. 2022;5(1):670. 35794204 10.1038/s42003-022-03610-7 PMC9259685

[43] The Global Parkinson’s Genetics Program. Code Reposistory. https://github.com/GP2code.

[44] Wang K, Li M, Hakonarson H. ANNOVAR: functional annotation of genetic variants from high-throughput sequencing data. Nucleic Acids Res. 2010;38(16):e164. 20601685 10.1093/nar/gkq603 PMC2938201

[45] Robinson JT, Thorvaldsdóttir H, Winckler W, Guttman M, Lander ES, Getz G, et al. Integrative genomics viewer. Nat Biotechnol. 2011;29(1):24–6. 21221095 10.1038/nbt.1754 PMC3346182

[46] Harris PA, Taylor R, Thielke R, Payne J, Gonzalez N, Conde JG. Research electronic data capture (REDCap)--a metadata-driven methodology and workflow process for providing translational research informatics support. J Biomed Inf. 2009;42(2):377–81. 10.1016/j.jbi.2008.08.010 PMC2700030 18929686

[47] Accelerating Medicines Partnership Parkinson’s Disease. (AMP-PD). https://amp-pd.org/.

[48] Chang CC, Chow CC, Tellier LC, Vattikuti S, Purcell SM, Lee JJ. Second-generation PLINK: rising to the challenge of larger and richer datasets. GigaScience. 2015;4(1):s13742-015. 10.1186/s13742-015-0047-8 PMC4342193 25722852

[49] Team R, RStudio. Integrated Development for R. RStudio, PBC, Boston, MA URL. http://www.rstudio.com/.

[50] LD Score Regression. https://github.com/bulik/ldsc.

[51] Terra.bio. Available from: https://app.terra.bio/#.

[52] Open Targets. https://www.opentargets.org/.

[53] Brumm MC, Pierz KA, Lafontant DE, Caspell-Garcia C, Coffey CS, Siderowf A, et al. Updated percentiles for the University of Pennsylvania smell identification test in adults 50 years of age and older. Neurology. 2023;100:e691–701. 10.1212/WNL.0000000000207077 PMC10115503 36849448

[54] Doty RL, Shaman P, Kimmelman CP, Dann MS. University of Pennsylvania smell identification test: a rapid quantitative olfactory function test for the clinic. Laryngoscope. 1984;94(2 Pt 1):176–8. 6694486 10.1288/00005537-198402000-00004

[55] Bryc K, Durand EY, Macpherson JM, Reich D, Mountain JL. The genetic ancestry of African americans, latinos, and European americans across the United States. Am J Hum Genet. 2015;96(1):37–53. 25529636 10.1016/j.ajhg.2014.11.010 PMC4289685

[56] Adler CH, Beach TG, Hentz JG, Shill HA, Caviness JN, Driver-Dunckley E, et al. Low clinical diagnostic accuracy of early vs advanced Parkinson disease: clinicopathologic study. Neurology. 2014;83(5):406–12. 24975862 10.1212/WNL.0000000000000641 PMC4132570

[57] Virameteekul S, Revesz T, Jaunmuktane Z, Warner TT, De Pablo-Fernández E. Clinical diagnostic accuracy of Parkinson’s Disease: where do we stand? Mov Disord. 2023;38(4):558–66. 36602274 10.1002/mds.29317

[58] Coughlin DG, Irwin DJ. Fluid and biopsy based biomarkers in Parkinson’s Disease. Neurotherapeutics. 2023;20(4):932–54. 37138160 10.1007/s13311-023-01379-z PMC10457253

[59] Schneider SA, Alcalay RN. Neuropathology of genetic synucleinopathies with parkinsonism: review of the literature. Mov Disord. 2017;32(11):1504–23. 29124790 10.1002/mds.27193 PMC5726430

